# Production of the Novel Two-Peptide Lantibiotic Lichenicidin by *Bacillus licheniformis* DSM 13

**DOI:** 10.1371/journal.pone.0006788

**Published:** 2009-08-26

**Authors:** Jasmin Dischinger, Michaele Josten, Christiane Szekat, Hans-Georg Sahl, Gabriele Bierbaum

**Affiliations:** Institute of Medical Microbiology, Immunology and Parasitology (IMMIP), University of Bonn, Bonn, Germany; The Research Institute for Children at Children's Hospital New Orleans, United States of America

## Abstract

**Background:**

Lantibiotics are small microbial peptide antibiotics that are characterized by the presence of the thioether amino acids lanthionine and methyllanthionine. Lantibiotics possess structural genes which encode inactive prepeptides. During maturation, the prepeptide undergoes posttranslational modifications including the introduction of rare amino acids as lanthionine and methyllanthione as well as the proteolytic removal of the leader. The structural gene (*lanA*) as well as the other genes which are involved in lantibiotic modification (*lanM*, *lanB, lanC*, *lanP*), regulation (*lanR*, *lanK*), export (*lanT(P)*) and immunity (*lanEFG*) are organized in biosynthetic gene clusters.

**Methodology/Principal Findings:**

Sequence comparisons in the NCBI database showed that *Bacillus licheniformis* DSM 13 harbours a putative lantibiotic gene cluster which comprises two structural genes (*licA1*, *licA2*) and two modification enzymes (*licM1*, *licM2*) in addition to 10 ORFs that show sequence similarities to proteins involved in lantibiotic production. A heat labile antimicrobial activity was detected in the culture supernatant and a heat stabile activity was present in the isopropanol cell wash extract of this strain. In agar well diffusion assays both fractions exhibited slightly different activity spectra against Gram-positive bacteria. In order to demonstrate the connection between the lantibiotic gene cluster and one of the antibacterial activities, two *Bacillus licheniformis* DSM 13 mutant strains harbouring insertions in the structural genes of the modification enzymes *licM1* and *licM2* were constructed. These strains were characterized by a loss of activity in the isopropanol extract and substractive MALDI-TOF predicted masses of 3020.6 Da and 3250.6 Da for the active peptides.

**Conclusions/Significance:**

In conclusion, *B. licheniformis* DSM 13 produces an antimicrobial substance that represents the two-peptide lantibiotic lichenicidin and that shows activity against a wide range of Gram-positive bacteria including methicillin resistant *Staphylococcus aureus* strains.

## Introduction

Lantibiotics (*lant*hionine containing ant*ibiotics*) are gene encoded, ribosomally synthesized antimicrobial peptides. They are characterized by extensive posttranslational modification reactions, which result in the formation of the unique thioether amino acids lanthionine and methyllanthionine as well as the dehydrated amino acids didehydroalanine (Dha) and didehydrobutyrine (Dhb) (for a current review see [1], [2]).

According to a recently proposed classification scheme, there are three main lantibiotic subgroups [2]. Type I lantibiotics comprise the linear peptides that are modified by LanB and LanC enzymes. Nisin and Pep5 are typical members of this class. Type II lantibiotics are globular peptides with the prototype lantibiotics mersacidin and cinnamycin that are modified by LanM enzymes. The type III lantibiotics SapT and SapB constitute an emerging group of lantibiotics that has mainly morphogenetic functions and displays very limited antibiotic activities [3], [4]. A further classification of lantibiotics can be based upon their mode of action: Some lantibiotics such as mersacidin [5] and planosporicin [6] bind to lipid II and thereby inhibit cell wall biosynthesis in sensitive bacteria. Other peptides attack the bacterial membrane by pore formation as e. g. Pep5 [7]. A third group is characterized by an ingenious double mode of action principle that combines inhibition of peptidoglycan biosynthesis by binding and dislocation of lipid II with pore formation in bacterial membranes. Both functions can be combined in a single molecule (e. g. nisin or epidermin) [8] or can be implemented in a combination of two functionally specialized peptides as shown for lacticin 3147 that is produced by *Lactococcus lactis*. In such two-peptide lantibiotics, the globular α-peptide with homology to mersacidin binds to lipid II and then forms a complex with the elongated β-peptide. Subsequently the β-peptide forms a pore in the bacterial membrane [9], [10].

In contrast to other peptide antibiotics, lantibiotics are gene encoded [1], [2]. The structural genes are usually found in biosynthetic gene clusters and encode prepeptides that consist of an N-terminal leader sequence that is typically separated by a conserved G(G/A/S) cleavage site from the propeptide, which is the precursor of the mature lantibiotic. The propeptide contains Ser, Thr and Cys residues that are later modified to yield the lanthionine and methyllanthionine residues. The enzymes involved in these modification reactions are either LanM enzymes, that catalyse both reactions (dehydration of the hydroxy amino acids and cyclization), or a combination of a LanB enzyme (dehydration of Ser and Thr residues) and a LanC enzyme (thioether formation) is present. Furthermore, many lantibiotic gene clusters contain a GG-leader peptidase that is the N-terminal domain of an ABC-type transporter. This protein functions in removal of the leader sequence concomitantly to the export of the lantibiotic. In addition, all lantibiotic gene clusters encode proteins that protect the producer strain against the antimicrobial effect of the lantibiotic, the so-called immunity proteins. Very often, especially with the lipid II binding lantibiotics, the immunity proteins are exporters that prevent binding of the lantibiotic to the bacterial membrane; however, membrane-associated peptides or cell wall bound proteins have also been discovered. Regulatory proteins are encoded in some but not all biosynthetic gene clusters [1], [2].

We performed a data base search for new lantibiotic gene clusters based on the conserved structure of the enzymes that are involved in the posttranslational modification procedures. To this end BLASTP at the NCBI database was used to find homologues of the mersacidin modification enzyme MrsM [11], the Pep5 modification enzyme PepB [12] and the cinnamycin modifying enzyme CinM [13] in other microorganisms, thus covering the main lantibiotic subgroups.

Among many other gene clusters, the blast yielded a biosynthetic gene cluster in *Bacillus licheniformis* DSM 13 (ATCC 14580) that encodes a lantibiotic prepeptide with apparent homology to mersacidin [14], which is a lantibiotic with promising anti-*Staphylococcus* activity [15]. For this reason, the antibacterial activity formed by *B. licheniformis* DSM 13 was chosen for further studies, especially as the absence of further polyketide (*pks*), pliastin (*pps*) and the bacitracin biosynthesis operons was reported in this genome. In contrast, the biosynthesis genes for lichenysin, which is a hemolytic surfactin-like toxin with activity against Gram-positive and Gram-negative bacteria [16] and eukaryotic cells are present [17], [18], however, failure of the strain to produce this substance had been reported earlier [19]. Therefore, no further production of antibiotics was expected. Here we report the identification and antibacterial activity of the new two-peptide lantibiotic lichenicidin and the antibacterial spectrum of an unanticipated substance that is present in the culture supernatant.

## Results

### A new lantibiotic gene cluster

The type strain of *B. licheniformis* has been deposited in several strain collections and the isolate of the American type culture collection (ATCC 14580) as well as the isolate of the DSMZ have been sequenced (DSM 13: GenBank accession No. AE017333 [17]) (ATCC 14580: GenBank accession CP000002 [18]). In the course of annotating both genomes, a putative lantibiotic structural gene had been noted and had been named lichenicidin [18] or had been annotated as “putative lantibiotic mersacidin precursor“ [17], respectively. Here we adopt the locus tags of the strain DSM 13 that was used in our experiments.

**Figure 1:**
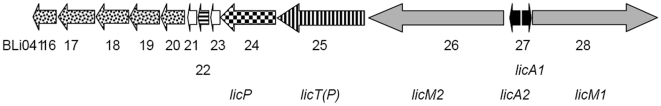
The lichenicidin gene cluster. The genes of the prepeptides are black; the genes of the two modification enzymes are grey. The other genes are marked with the following patterns: processing transporter, vertical stripes; the peptidase gene, checkerboard pattern; the putative regulator gene, horizontal stripes; genes that might be involved in immunity and encode proteins that have similarity to transporters, dots. The numbers give the last two digits of the locus tags in the annotation of the *B. licheniformis* DSM 13 isolate. For the small white orfs, no functions could be assigned so far, however similar orfs are encoded in the vicinity of the haloduracin gene cluster.

The gene cluster of lichenicidin **(** **Fig. 1** **)** is located on both strands at the far end of the chromosome and covers bp 3938843 to 3953096 (BLi04116 to BLi04128) in a total of 4222748 bp. The GC content (45.1%) of the cluster is quite similar to the average GC content of the whole genome (46.2%) [17] and the gene cluster does not seem to be associated with any mobile element.

**Figure 2:**
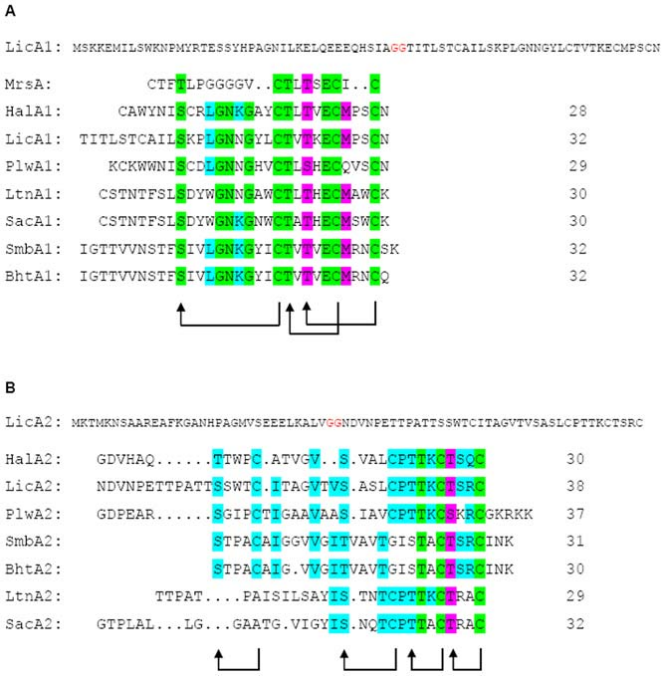
Amino acid alignments of two-peptide lantibiotics. (A) Amino acid sequence alignment of the LicA1 propeptide with the LanA1 propeptides of the two-peptide lantibiotics plantaricin (PlwA1, AAG02567), staphylococcin C55 (SacA1, BAB78438), lacticin 3147 (LtnA1, O87236), haloduracin (HalA1, BAB04173), BHT (BhtA1, AAZ76603) and Smb (SmbA1, BAD72777) and, for comparison, the propeptide of mersacidin (MrsA, Z47559). Amino acid identities of the LanA1 propeptides are highlighted in green (100%), pink (75%) and blue (35%). The thioether bridging pattern represents that of the Halα and Plwα peptide. (B) Amino acid sequence alignment of the LicA2 propeptide with the Lanβ/LanA2 propeptides of the two-peptide lantibiotics plantaricin (PlwA2, AAG02566), staphylococcin C55 (SacA2, BAB78439), lacticin 3147 (LtnA2, O87237), haloduracin (HalA2, BAB04172), BHT (BhtA2, AAZ76602) and Smb (SmbA2, BAD72776). Amino acid identities are highlighted in green (100%), pink (75%) and blue (35%). The thioether bridging pattern represents that of the Halβ and Plwβ peptides.

Two genes, BLi04126 (34% amino acid sequence identity to HalM2 [20] and BLi04128 (34% identity to HalM1 [20], 33% identity to MrsM [11]) encode the modifying enzymes that were recognised during the data base search using MrsM. In between, one structural gene BLi04127 was annotated that encodes the lantibiotic prepeptide (see above) with similarity to mersacidin (40%) [14] and HalA1 (38%) [21], which belong to the class II lantibiotics as well as other α-peptides of two-peptide lantibiotics **(** **Fig. 2a** **)**. However, the presence of two modification enzymes indicates the presence of a second, dissimilar prepeptide as seen in the gene clusters of lacticin 3147 [22], haloduracin [20] and staphylococcin C55 [23]. Lantibiotic structural genes can be identified by Cys/Ser/Thr residues in the C-terminus of the prepeptides and their conserved cleavage sites. Indeed, upon closer analysis upstream of the structural gene a second structural gene, that had been overlooked during annotation in both available sequences, was found to be located 8 bp downstream of an AGGAGG Shine- Dalgarno sequence, encoding a 72 amino acid (aa) prepeptide (bp 3949269–3949514) and consisting of a leader of 34 aa and a propeptide of 38 aa. This prepeptide shows similarity to the haloduracin β-peptide HalA2 (52%) [21], i. e. a gene which encodes a peptide with an elongated structure; the similarities to the other β-peptides of two-peptide systems were lower, ranging from 11% (CylL_L_) [24] to 30% (LtnA2) [22] (**Fig. 2b**). The presence of this gene was also suggested in a recent publication [21]. Further downstream BLi04125 constitutes a transporter with a peptidase subunit and 49% similarity to HalT(P) [20], and BLi4124 encodes a protein with an N-terminal signal domain that shows similarity to a peptidase involved in processing of the lantibiotic cytolysin (CylA, 37%) and a peptidase encoded in the genome of *B. halodurans* C-125 (BH1491, 49%) [25]. BLi04123 is a small hypothetical protein. BLi04122 constitutes a helix-turn-helix protein that shows 60% similarity to a protein that is encoded near the haloduracin gene cluster (BH0460) and may be involved in regulation. BLi04121 is a small protein that also is similar (61%) to a protein encoded downstream of the haloduracin gene cluster (BH0459) [25] and harbors three transmembrane helices. The following orfs might be involved in immunity against the lantibiotic: BLi04120 is the ATP binding domain of an ABC transporter. BLi04119 does not show any sequence similarity to known proteins, however, six transmembrane sequences are predicted for this protein. BLi04118 shows low similarity to the MrsE protein of the mersacidin gene cluster (21%) [26] and likewise, a computer analysis shows six membrane spanning regions. The N-terminal two thirds of BLi04117 appear homologous to the bacitracin transporter of *B. licheniformis*, a protein that is able to prevent the binding of bacitracin to undecaprenyl-pyrophosphate [27], to the ATPase component of the CcmA multidrug resistance system and to the ATPase subunit of the haloduracin gene cluster HalF1 (35.8%) [20]. BLi04116 again is a transmembrane protein with six membrane spanning helices and similarity to BcrB (52%). The following gene downstream encodes a protein with similarity to the ferrochelatase HemH, indicating that the gene cluster ends here. Upstream the gene cluster is bordered by BLi04129, encoding a pectate lyase. In conclusion, this gene cluster represents 14 Orfs, with two lantibiotic structural genes, two modification enzymes, a peptidase, one regulator and probably two different immunity systems (an ABC transporter BLi04120-BLi04118 and a transporter with homology to the bacitracin transporter BLi4117/BLi4116). After a search of both annotated sequences for “free” gene designations we propose according to established lantibiotic gene nomenclature the following gene designations for the ORFs involved in biosynthesis of lichenicidin: *licA1* (BLi04127, the structural gene of the mature α-peptide Licα), *licA2* (the structural gene of the mature β-peptide Licβ); *licM1* (BLi04128 encoding the LanM that modifies LicA1), *licM2* (BLi04126 encoding the LanM modifying LicA2), *licT(P)* (BLi04125 encoding the processing transporter) and *licP* (BLi04124 encoding the peptidase).

### *B. licheniformis* produces at least two antibiotic substances

**Figure 3:**
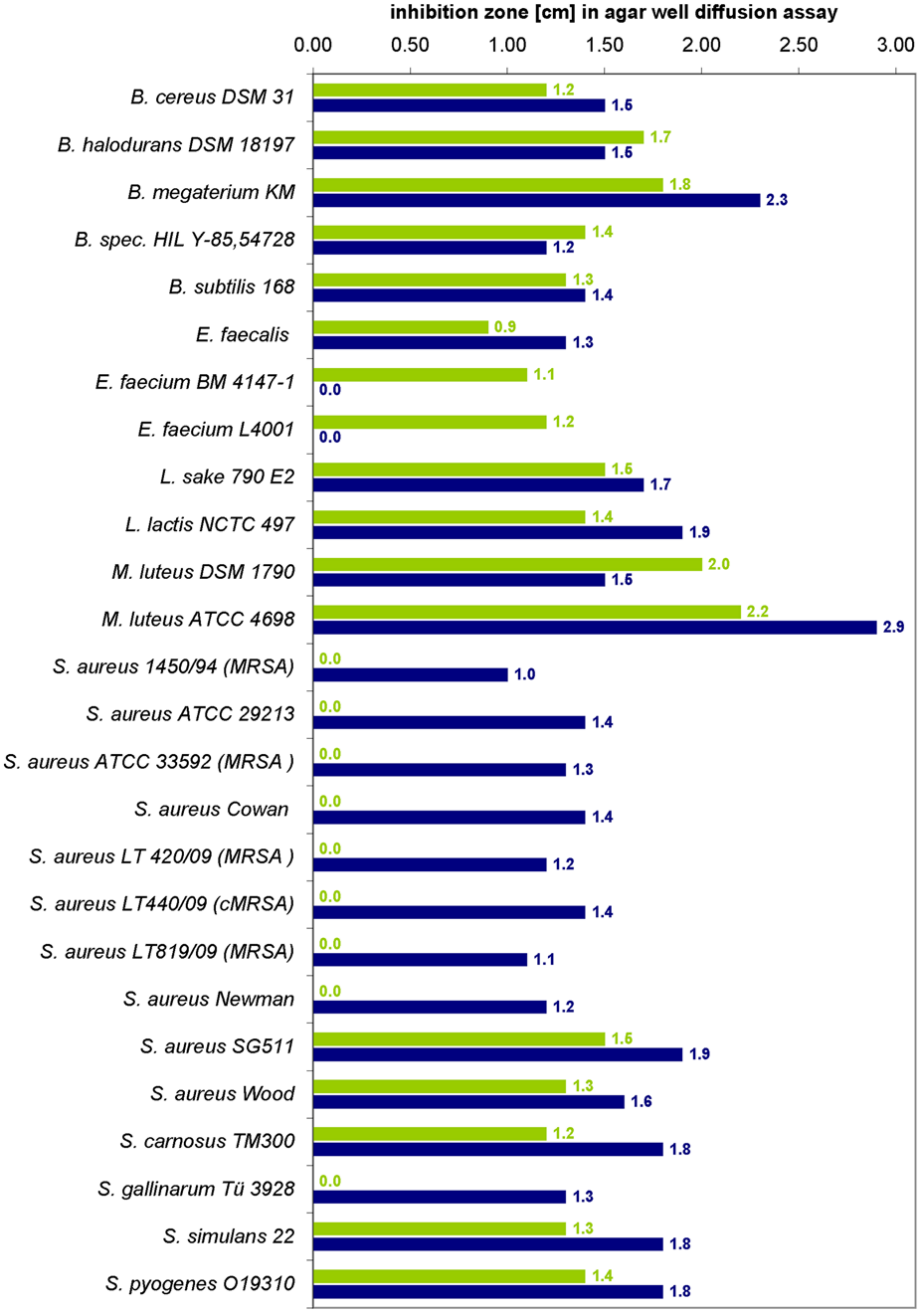
Agar well diffusion assays of the *B. licheniformis* DSM 13 culture supernatant and the isopropanol extract. The culture supernatant (green bars) as well as the isopropanol extract (blue bars) were active against Gram-positive bacteria but showed slightly different spectra of activity, indicating that supernatant and isopropanol cell wash extract contained different antibacterial compounds.

First production experiments and activity tests with crude supernatant in synthetic medium (2×BPM), tryptic soy both, lysogeny broth (LB) and Mueller Hinton broth confirmed that *B. licheniformis* DSM 13 indeed produced an antibacterial activity that affected the growth of Gram-positive bacteria as *Bacillus subtilis*, *Micrococcus luteus*, *Staphylococcus aureus*, *Streptococcus pyogenes*, *Staphylococcus simulans* and enterococci but neither caused hemolysis nor inhibited the growth of Gram-negative bacteria. Furthermore, the isopropanol wash extract of the cell pellet from 2×BPM was also non-hemolytic and showed antibacterial activity against Gram-positive bacteria including several methicillin resistant *Staphylococcus aureus* (MRSA) strains, however with a slightly different spectrum of activity, indicating that the culture supernatant and the isopropanol cell wash extract might contain different substances (**Fig. 3**). Gram-negative bacteria and eukaryotic cells were not affected (data not shown).

**Figure 4:**
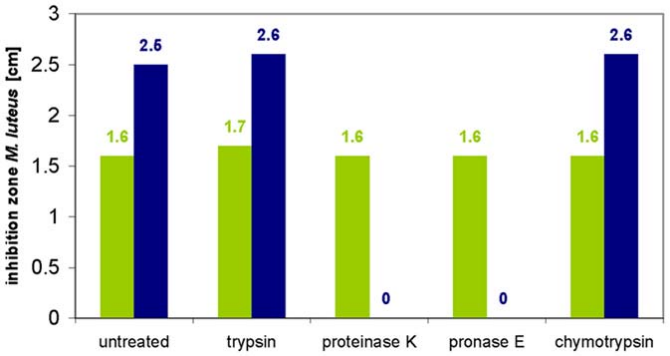
Stability assay: Treatment with proteases. No loss of both antimicrobial activities was detected after treatment with trypsin and chymotrypsin for 4 h for both extracts. However, incubation of the isopropanol cell wash extract (blue bar) with proteinase K and pronase E resulted in a total loss of activity against *M. luteus* while the antimicrobial activity of the culture supernant (green column) was unaffected by these proteases.

A further characterization of both active fractions showed that the supernatant contained a substance displaying only limited heat stability that was inactivated within two hours by 80°C and within 30 min by boiling, whereas the activity in the isopropanol extract was fully heat stable and withstood 4 h at 100°C without any losses. Both active fractions caused inhibition of growth of the indicator strain *M. luteus* in a deferred antagonism test at pH 1.5, 3 and 9, however, the supernatant did not show any antibacterial activity at pH 13. In contrast, the isopropanol cell extract was initially active but inactivated after 4 h at pH 13. Incubation in the presence of proteolytic enzymes (pronase E, proteinase K, trypsin, chymotrypsin) did not affect the antibacterial activity present in the supernatant. The isopropanol extract was also stable in the presence of trypsin and chymotrypsin, however, addition of proteinase K and pronase E led to inactivation within 4 h of incubation, indicating the proteinaceous nature of this substance **(** **Fig. 4** **)**. Cross resistance of *B. halodurans* C-125, which produces the closely related two-peptide lantibiotic haloduracin [21], was also tested, however, *B. halodurans* C-125 was sensitive to both fractions. The same result was obtained with the producer of mersacidin, *Bacillus spec*. HIL Y-85,54728 [28].

### Inactivation of the lantibiotic modification enzymes

**Figure 5:**
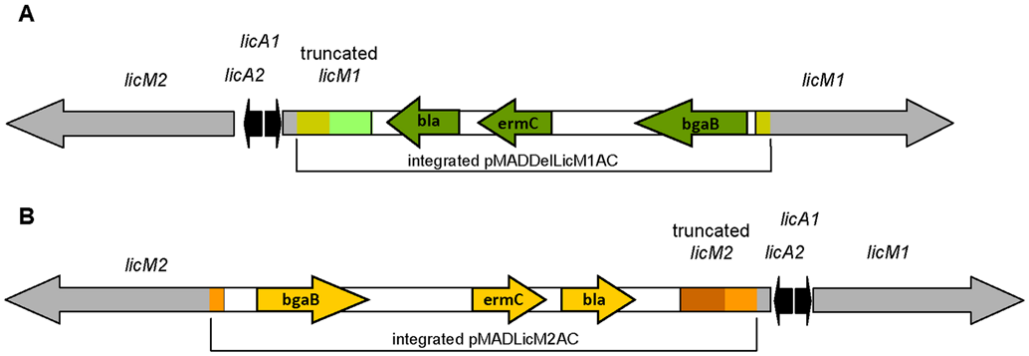
Gene inactivation of the lantibiotic modifications enzyme LicM1 (A) and LicM2 (B) by homologous recombination. Gene inactivations of the modifications enzymes LicM1 and LicM2 were performed by plasmid integration using the recombinant plasmids pMADDelLicM1AC and pMADLicM2AC. The resulting insertion mutants *B. licheniformis* LicM1INT (A) and LicM2INT (B) harbour two copies of the *lanM* genes within the lantibiotic gene cluster: a copy that is transcribed but is truncated and a second copy that does not dispose of promoter, Shine Dalgarno sequence and start codon. Genes that derive from the plasmid pMADDelLicM1AC are marked in green colours and those deriving from pMADLicM2AC are marked in orange and red.

In order to correlate the antimicrobial activity with the presence of the putative lantibiotic gene cluster, the biosynthetic enzymes LicM1 (BLi04128) and LicM2 (BLi04126) were both inactivated by insertion of a vector that carried two fragments of the gene employing the temperature sensitive plasmids pMADDelLicM1AC and pMADLicM2AC, respectively. Based on the vector pMAD, these plasmids harbor the *bgaB* gene (blue white selection), the *ermC* gene (erythromycin-resistance) and the 5′ region and 3′ regions of the genes *licM1* or *licM2* lacking the start and stop codons. Both plasmids were introduced in *B. licheniformis* MW3, a transformable strain of *B. licheniformis* DSM 13, since the producer strain is characterized by loss of natural competence and a very active restriction system [29]. Insertion mutants harboured two copies of the LanM enzyme in their genome. The transcribed copy of *licM1* which is located downstream of *licA1* harbours an internal deletion that leads to a loss of 718 aa. The second copy is located downstream of the *bga* gene on the opposite strand and does not dispose of promoter, Shine-Dalgarno sequence and start codon. The first copy of *licM2* leads to transcription of a protein with a C-terminal deletion of 873 aa and the second copy again cannot be transcribed. Furthermore, integration of the plasmid into BLi04126 was expected to exert a polar effect and inhibit transcription of the downstream genes of the gene cluster i. e. of the transporter and peptidase that are probably involved with export and processing of both peptides (**Fig. 5**).

**Figure 6:**
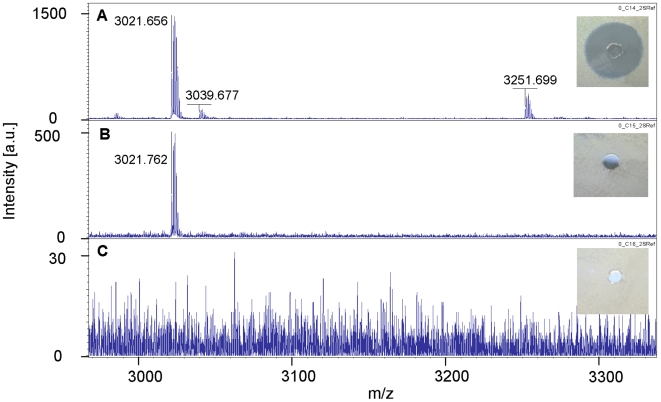
MALDI-TOF mass spectra of the *B. licheniformis* MW3 wild type (A) and its insertion mutans LicM1INT (B) and LicM2INT (C). The isopropanol extracts of the insertion mutants *B. licheniformis* LicM1INT and LicM2INT were characterized by the loss of activity against *M. luteus*. MALDI-TOF spectra of these isopropanol extracts in comparison to the wildtype (A) showed the loss of a peak at 3251 Da in the case of the LicM1 insertion mutant (B) indicating that this peak represents the protonated form of the active Licα peptide. The insertion of a plasmid into the gene of the modification enzyme LicM2 inactivated this enzyme and most probably exerted a polar effect on the downstream genes, thus affecting production of both peptides. This mutant did not produce the Licα peptide and is further characterized by the absence of a 3021 Da peak, which might represent the protonated form of the mature Licβ peptide, harboring 12 dehydrated residues (C). In some cultivations a further peak of 3039 Da was observed, which probably denotes a Licβ peptide with only 11 of 15 possible dehydrations.

After inactivating the modifying enzyme of LicA1, the LicM1 insertion mutant was characterized by a loss of activity of the isopropanol cell wash extract against *M. luteus*. In contrast, the activity of the culture supernatant was not affected. MALDI-TOF mass spectrometry showed the loss of a peak at 3251.7 Da (*m*+1H)^1+^ in the isopropanol wash extract of this mutant (**Fig. 6a** **/b**). After removal of the leader at the conserved GG cleavage site, the unmodified propeptide LicA1 possesses a calculated molecular mass of 3376.9 Da. Therefore, the missing peak might correspond to the mature Licα where seven of eight possible dehydrations have taken place (−126 Da) during the posttranslational modification reactions.

Furthermore, all antibacterial activity in the isopropanol cell wash extract was also lost in the LicM2 integration mutant. The antibacterial activity in the culture supernatant remained unaffected, indicating that this activity is definitely not related to the lantibiotic gene cluster. The MALDI-TOF spectrum of the cell wash extract lacked the peaks [(*m*+1H)^1+^] at 3251.7 Da as well as at 3021.7 and a minor peak at 3039.6 Da that was present in some preparations indicating a correlation of all three masses to the lichenicidin gene cluster (**Fig. 6c**). The calculated mass of the unmodified propeptide LicA2 amounts to 3905.3 Da. A mass of 3020.6 Da can be predicted for a Licβ peptide that has lost the six N-terminal amino acids by proteolytic processing and harbors 12 dehydrated residues and three hydroxy amino acids. A mass of 3038.6 Da would correspond to a peptide with only 11 dehydrations. The N-terminal processing also leads to the loss of two negatively charged residues (Asp and Glu), resulting in a positive net charge for Licβ, which is characteristic for membrane active peptides. None of the three masses was present in the spectra of the culture supernatant.

**Figure 7:**
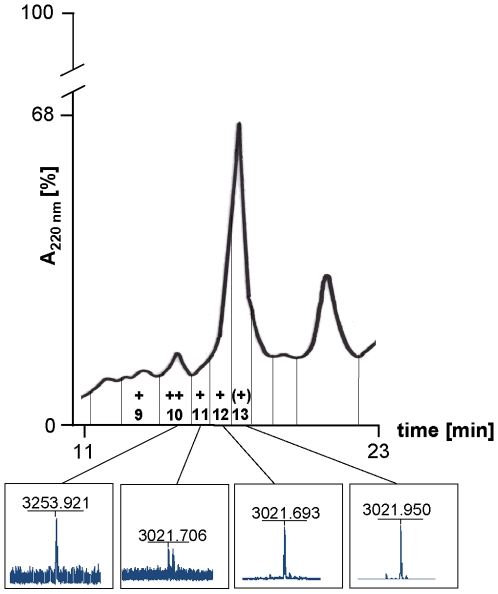
HPLC chromatogram of the *Bacillus licheniformis* isopropanol extract. The isopropanol extract was applied to a POROS RP-HPLC column and eluted in a gradient of 20% to 55% acetronitrile (containing 0.1% TFA). Maldi-TOF analysis of active fractions [+ medium activity, ++ strong activity and (+) poor activity] showed the presence of masses representing the Licα peptide or the Licβ peptide.

**Figure 8:**
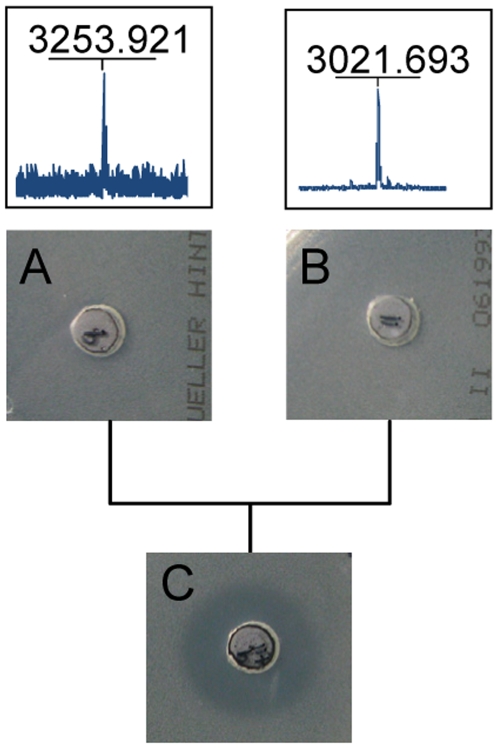
Synergistic effect of fractions containing the Licα and Licβ peptides. 25 µl of HPLC fractions that contained the Licα peptide or the Licβ peptide as seen in mass spectrometry were tested separately and in combination against *M. luteus*. Only the combination of both peptides showed an antimicrobial activity indicating that Licα and Licβ are required for an optimal effect.

In order to show that the lost masses corresponded to the antibacterial activity, the isopropanol cell wash extract was fractionated by HPLC on a Poros column and the masses of the active fractions were determined. As shown in **Fig. 7** the masses for Licα (fraction 9) and Licβ (fraction 10–13) were associated with the active fractions confirming that these peptides represent the two-peptide lantibiotic lichenicidin. A combination of fraction 9 and fraction 11 (25 µl of each fraction) showed enhanced activity, indicating a synergistic action of both peptides (**Fig. 8**).

**Figure 9:**
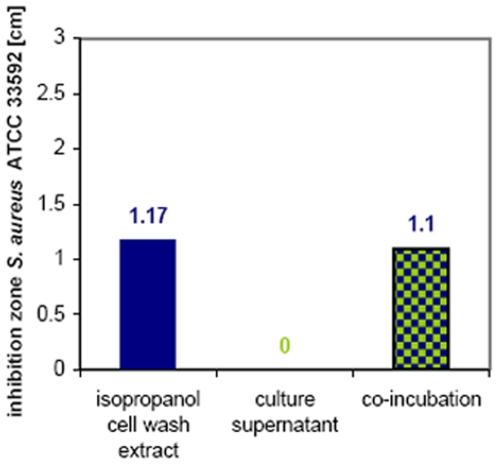
Co-incubation of the isopropanol cell wash extract and the culture supernatant. In agar well diffusion assays, the isopropanol extract (25 µl) showed an antimicrobial activity against *S. aureus* ATCC 33592 (blue bar) while the culture supernantant was inactive (see also Fig. 3). In order to test protease stability of the isopropanol extract, 25 µl of extract were mixed with 25 µl of culture supernatant (blue-green patterned bar), incubated for 2 h and then tested by agar diffusion. The co-incubation of both extracts had no effect on the activity of the isopropanol extract, indicating that the antimicrobial substance is stable against the proteases excreted by the producer strain.

Because the lantibiotic seemed to be present only in the cell wall associated form, culture supernatant, that contained protease activity as determined on skim milk agar plates, was incubated with the isopropanol wash extract in order to test the protease stability of lichenicidin. After 2 hours of incubation, the activity against *S. aureus* ATCC 33592 or *S. gallinarum* (data not shown) was not diminished, indicating that the peptides are indeed protease resistant (**Fig. 9**).

### Antibiotic efficacy of lichenicidin against MRSA

In order to compare the antibiotic efficacy of lichenicidin against MRSA strains to that other lantibiotics, we tested solutions containing lichenicidin, mersacidin, Pep5 and nisin against *S. aureus* SG511 (sensitive laboratory strain - MSSA), *S. aureus* ATCC 33592 (MRSA) and *S. aureus* LT440/09 (cMRSA). All lantibiotics had to be employed at higher concentrations to inhibit growth of the MRSA strains than for the MSSA; the minimum inhibitory concentrations on agar plates were 1.83±0.41-fold (ATCC 33592) or 2.67±1.03-fold (LT440/09) higher for nisin, 4.8±1.6-fold (ATCC 33592) and 4.7±1.6-fold (LT440/09) higher for mersacidin, 8-fold (ATCC 33592) and 7.33±1.63-fold (LT440/09) higher for lichenicidin and 18.7±6.5-fold (LT440/09) or 26.6±8.26-fold (ATCC 33592) higher for Pep5 than the minimum inhibitory concentrations against *S. aureus* SG511.

## Discussion

Potential applications for lantibiotics are food conservation and novel therapeutics. An obstacle that has hampered the medical application of many lantibiotics is their missing resistance to the proteases; this is especially characteristic for the elongated pore-forming peptides like nisin [30], epidermin [31] and Pep5 [32]. These peptides possess a hinge region which is essential for pore-formation and which is susceptible to the attack of proteases like chymotrypsin or trypsin. Stabilization of Pep5 by a further ring structure prevented chymotrypsin activity, however, the antibacterial activity was also decreased [32]. In contrast, those peptides that are characterized by formation of intertwined rings and do not have elongated linear stretches show higher protease resistance as for example mersacidin. One reason that *B. licheniformis* DSM 13 was chosen for further investigations is that it is most prominent in protease production. Seven protease genes are annotated in the genome of this strain (BLi00340, BLi04019; BLi01123; BLi02863; BLi02862, BLi01109, BLi01909) among them Subtilisin Carlsberg, a protease that is employed in detergents [17]. Therefore, the antibacterial activity excreted by this strain was expected to show higher protease resistance than lantibiotics produced by members of the genera *Staphylococcus* and *Lactococcus*.

Closer inspection indicated that this gene cluster might encode a two-peptide lantibiotic with similarity to haloduracin. So far seven two-peptide antibiotics have been described [21], [33]–[38], for a review see [39]. With the exception of the Lys in the third ring (and this position is taken by His in some other peptides), all amino acids found in Licα are present in at least one other peptide of the group (Fig. 2a). The masses indicate that one residue of LicA1 escapes dehydration and this might be the residue that is not dehydrated in haloduracin [40], the Ser in the C-terminal ring structure. In addition, the location of the ring forming and other essential amino acids, i. e. the LanA1 motif, is conserved for the terminal three rings, indicating that the C-terminal ring structure of Licα might be identical to that of the other α-peptides with a solved structure (lacticin 3147 [41] and haloduracin [40]). This close similarity does not extend to the N-terminus of the peptide, where, just as in mersacidin, a fourth (Me)Lan might be formed, since a Cys and several didehydro amino acids are present.

As with LicA1, the LanA2 motif is conserved in LicA2 (Fig. 2b), indicating that the three C-terminal rings might be formed as in haloduracin [21]. The N-terminus again deviates from the other peptides and contains several hydroxy amino acids. In addition, the Licβ peptides are characterized by a mass that is consistent with 11 and 12 of 15 possible dehydrations and an N-terminal processing of six amino acids. An N-terminal processing has also been demonstrated for Halβ [40] which is characterized by an N-terminal disulfide ring structure. The protease encoded by BLi4124 contains a secretion signal and might well be involved here. The loss of six N-terminal amino acids places a Thr residue at the N-terminus of Licβ, thereby enabling an N-terminal MeLan ring, which would inhibit any further protease activity. The resistance of the antibacterial activity in the extract to digest with chymotrypsin strongly indicates the presence of such a fourth ring structure in the N-terminus since there is a cleavage site (TTPATTSSW↓TC) located in this area. However, the N-terminus contains six hydroxy amino acids that might serve for the formation of an N-terminal ring structure, so that further structural analysis is required in order to judge the size of the ring. A possible proteinase K cleavage site is located in the linear region of the peptide (ITA↓GVTV) and might account for the loss of activity seen with this enzyme.

In conclusion, this study shows that *B. licheniformis* DSM 13 produces a novel two-peptide lantibiotic that is associated with the cell surface and that shows stability against trypsin, chymotrypsin and the proteases of the culture supernatant of the producer. Remarkably, the antibacterial spectrum of lichenicidin showed a good activity against a range of MRSA strains that cause problematic infections in the hospital setting. The test panel included clinical isolates of epidemic strains that are currently widespread in Germany. A second substance with activity against Gram-positive bacteria is present in the culture supernatant. Lack of hemolytic activity and the activity spectrum indicate that this substance does not represent lichenysin [16], so that its origin in the genomic context remains unclear. Lichenysin itself was not produced although the biosynthesis genes are present in the strain. The failure of lichenysin production here [19] might be due to the fact, that transcription of the biosynthetic machinery of lichenysin is activated by the phosphorylated form of ComA [42]. However, the gene of the kinase ComP necessary for activation of ComA [43] has suffered insertion of an IS element in *B. licheniformis* DSM 13 [18].

The other open question regards the localization of lichenicidin in the cell wash extract. A location in the cell wall of the producer strain has also been described for the haloduracin [20] and lacticin peptides. For the lacticin peptides, mutant studies indicated that the LtnA2 peptide might be tethered to the cell wall via the LtnA1 peptide [44]. Furthermore, the diffusion of the lichenicidin peptides might be restricted by capsule formation: Both mature peptides carry a positive net charge (Licα +1, Licβ +2). *B. licheniformis* is able to produce a glutamyl polypeptide capsule [18]. Incubation of another strain of this species, *B. licheniformis* DSM 641, on nisin containing agar plates led to selection of nisin resistant mutants with increased capsule formation (Bierbaum, unpublished results), which indicates that the capsule might indeed inhibit the diffusion of or bind cationic lantibiotics. However, such a location seems inappropriate, considering that the overwhelming majority of lantibiotics is regarded as bacteriocins. These molecules function in the defense of the producer strain. To this end, the peptides would have to be present in the medium. On the other hand, the morphogenetic peptides of *Streptomyces* are lantibiotics, too [45], and the genomic sequences that recently appeared in the databases have shown that lantibiotic biosynthesis genes are widespread among bacteria. In this context, these results might indicate that other functions for lantibiotics in the morphogenesis or metabolism of the producer strain may be possible and that we are only just starting to learn about why bacteria produce these fascinating peptides.

## Materials and Methods

### Bacterial strains, culture media, growth conditions and detection of antibacterial activity

The producer strain *Bacillus licheniformis* DSM 13 was purchased from the German Collection of Microorganisms and Cell Cultures (DSMZ, Braunschweig, Germany). A transformable variant of *B. licheniformis* DSM 13, the MW3 strain, was kindly provided by Prof. Meinhardt (Institute of Molecular Microbiology and Biotechnology, University of Münster) [29]. Both *B. licheniformis* strains were grown aerobically in tryptic soy broth or on tryptic soy agar (Oxoid, Hampshire, Great Britain). For the detection of lantibiotic production the producer and its mutants were cultured in two-fold *Bacillus* production medium (2×BPM) [46]. *Escherichia coli* SCS110 was used as intermediate cloning host and was cultured in Lysogeny Broth (LB) medium at 37°C. For antibiotic activity testing the following indicator strains were used: *Bacillus cereus* DSM 31, *Bacillus halodurans* DSM 18197, *Bacillus megaterium* KM (ATCC 13632), *Bacillus subtilis* 168 (DSM 402), *Bacillus spec*. HIL Y-85,54728 [28], *Enterococcus faecium* BM 4147–1 [47], *Enterococcus faecium* L4001, *Lactobacillus sake* 790 E2, *Lactococcus lactis* NCTC 497, *Micrococcus luteus* DSM 1790, *Micrococcus luteus* ATCC 4698, *Staphylococcus aureus* ATCC 33592 (MRSA), *S. aureus* ATCC 29213 (MSSA), *S. aureus* 1450/94 (Northern German epidemic strain, German Reference Centre for Staphylococci, Wernigerode, Germany), S. *aureus* Cowan (ATCC 12598), *S. aureus* Newman (NCTC 8178), *S. aureus* SG511 [48], S. *aureus* Wood 46 (ATCC 10832), *Staphylococcus carnosus* TM300 [49], *Staphylococcus gallinarum* Tü 3928 [50], *Staphylococcus saprophyticus* DSM 20229, *Staphylococcus simulans* 22 [51], and the following clinical isolates: *S. aureus* LT440/09 (community acquired MRSA), *S. aureus* LT420/09 (MRSA), *S. aureus* LT819/09 (MRSA, Rhine-Hessen epidemic strain), *Enterococcus faecalis, Streptococcus agalactiae* and *Streptococcus pyogenes* O-19310. For stock preparation, the cells were cultivated overnight, mixed with sterile glycerol (final concentration of 50% v/v) and stored at −70°C.

For selective media, antibiotics (Sigma-Aldrich, Taufkirchen, Germany) were added at the following concentrations: ampicillin (40 mg/l) for *E. coli* and erythromycin (25 mg/l) for *B. licheniformis* strains.

For the comparison of the antibacterial efficacy of lichenicidin to other lantibiotics, Pep5 [52] and nisin [53] were purified as described previously, and mersacidin was obtained from Sanofi-Aventis (Frankfurt, Germany). Serial dilutions of the lantibiotics and the isopropanol wash extract were prepared in half-concentrated Müller-Hinton broth. Fresh colonies of all indicator organisms were used to prepare cell suspensions of about 5×10^6^ colony forming units per ml which were employed to inoculate Müller Hinton agar plates. Ten µl of the lantibiotic solutions were spotted onto the indicator plates. The highest dilution yielding a clear inhibition zone on the lawn was detected by visual inspection after incubation overnight.

### Detection of antimicrobial and protease activity

The detection of antimicrobial activity was performed by agar well diffusion assays on Müller-Hinton agar II plates (Difco^TM^, Detroit, USA) seeded with an indicator strain in wells with a diameter of 7 mm employing 50 µl of sample unless indicated differently [54]. After incubation at 37°C overnight, the growth inhibition zones were measured.

The protease activity in the cell-free culture supernatants was detected on skim milk agar plates (3 g/l yeast extract, 5 g/l peptone, 2.5 g/l skim milk powder, 15 g/l agar, pH 7.2). 50 µl of the culture supernatant were applied to wells (7 mm). After incubation at 37°C overnight, the plates were inspected for the formation of clear zones.

### DNA and Plasmid preparation

Genomic DNA was prepared using the PrestoSpinD Kit according to the recommendations of the supplier (Molzym, Bremen, Germany). Plasmid DNA was isolated using the GeneJET^TM^ Plasmid Miniprep Kit (Fermentas, St. Leon-Rot, Germany).

### Inactivation of the lantibiotic modifying enzymes LicM1 and LicM2

The inactivation of the lantibiotic modifying enzymes LicM1 and LicM2 was performed by plasmid integration using the thermosensitive shuttle vector pMAD [55]. In pMADDelLicM1AC the vector carries the 5′ and 3′ parts (each 500 bp) of the gene *bli04128* (*licM1*) lacking the start and the stop codon. The fragments were generated via PCR using the Phusion Polymerase (2 u/µl, NEB, Beverly, USA) and the oligonucleotide pair forΔLicM1fragmentA and revΔLicM1fragmentA for the amplification of the 5′ part and the primer pair forΔLicM1fragmentC and revΔLicM1fragmentC for the 3′ part of the *licM1* gene (table 1). The PCR products were purified using the MinElute PCR Extraction Kit (QIAGEN, Hilden, Germany) and digested with the FASTdigest^TM^ enzymes *Mlu*I, *Bgl*II and *BamH*I following the manufacturers' instructions (Fermentas, St. Leon-Rot, Germany). Both fragments were ligated (T4 ligase, 5 u/µl, Fermentas, St. Leon-Rot, Germany) in tandem into the corresponding restriction sites of pMAD. The resulting vector pMADDelLicM1AC was introduced into *E. coli* SCS110 as intermediate cloning host by electroporation (Bio-Rad, Munich, Germany).

**Table 1:** Oligonucleotides used in this study.

Target gene	Oligonucleotide	Sequence
*licM1* (5′ part)	forΔLicM1fragmentA	5′ GATGGATCCAATGAAAAATCCGCCG 3′
	revΔLicM1fragmentA	5′ TCGACGCGTTCGCCCTTCAATTGATCTTC 3′
*licM2* (3′ part)	forΔLicM1fragmentC	5′ TTCACGCGTCCTGGCATTTGAAAAGCAGC 3′
	revΔLicM1fragmentC	5′ TTGAGATCTAAACACGTTTTCTCTTTTAAAAGC 3′
*licM2* (5′ part)	forDelLicM2A	5′ ATTACGCGTTGTGATAAGTGTTCTCGTCGC 3′
	revDelLicM2A	5′ GCACCATGGGTTTTCTTCGCCAAAGGGATG 3′
*licM2* (3′ part)	forDelLicM2C	5′ ATCGGATCCGCCCGTCGGA ATATCGAGA 3′
	revDelLicM2C	5′ CGGACGCGTTGATACACGATGACAGCTGC 3′
mcs of pMAD	pMADmcs2neu	5′ GAAGCGAGAAGAATCATAATG 3′
*licM1*	forhomRecLicM1	5′ TGAATCTTCTTATCATCCAGC 3′
	revhomRecLicM1	5′ GCATTTGGATGAAGGTCTTTC 3′
*licM2*	forhomRecLicM2	5′ CCAACAACTAAGTGTACAAGC 3′
	revhomRecLicM2	5′ ACCGTCCGGCTACCATCG 3′

According to this protocol, a second derivative of pMAD called pMADLicM2AC was generated for the inactivation of the LicM2 enzyme. The 3′ and 5′ parts of the gene *bli04126* were amplified using the primer pairs forDelLicM2A and revDelLicM2A and forDelLicM2C and revDelLicM2C (see table 1). Integrity of the plasmids was confirmed by sequencing of the inserts (Sequiserve, Vaterstetten, Germany). The recombinant vectors pMADDelLicM1AC and pMADDelLicM2AC were then introduced in *B. licheniformis* MW3 via protoplast transformation [29] and transformants were selected at 30°C. Recombinants that had inserted the plasmids into the genomes were selected after cultivation overnight at the nonpermissive temperature (45°C) in TSB (EM 25 mg/l) and subsequent plating on TSA agar plates (EM 25 mg/l). The insertions of the vectors into the target genes were confirmed by PCR using primers that anneal within the genome sequence and flank the target genes (forhomRecLicM1, revhomRecLicM1, forhomRecLicM2, revhomRecLicM2) as well as a primer that anneals within the vector sequence (pMADmcs2neu) (Table 1). The resulting insertion strains were named *B. licheniformis* MW3 LicM1INT and LicM2INT.

### Production and peptide preparation

For lantibiotic production *Bacillus licheniformis* DSM 13 and MW3 were cultured in 50 ml 2×BPM at 37°C with agitation. The production experiments with the *Bacillus licheniformis* MW3 insertion mutants LicM1INT and LicM2INT were performed at 45°C. After 48 hours of incubation the cells were pelleted by centrifugation at 10,000×g and 4°C for 30 minutes. For further analysis the culture supernatant was sterilized by filtration and stored at −20°C. The cell pellet was washed with 35 ml 70% isopropanol (adjusted to pH 2 with HCl) and incubated at 4°C for four hours under stirring [44]. The cells were removed by centrifugation and the supernatant was sterilized by filtration and stored at −20°C. For HPLC analysis, the isopropanol was removed by rotary evaporation (Rotavapor Re11, Essen, Germany). Two ml of extract containing 0.1% trifluoroacetic acid (TFA, Sigma-Aldrich, Taufkirchen, Germany) was applied to a Poros RP-HPLC-column (10R2, 100×4.6 mm, Perseptive Biosystems, Freiburg, Germany) and eluted in a gradient of 20% to 55% acetonitrile (containing 0.1% TFA). The peaks were detected measuring the absorbance at 230 and 260 nm. The fractions were collected and assayed for the antimicrobial activity against *M. luteus* ATCC 4698 in agar well diffusion assays as well as analyzed by MALDI-TOF spectrometry.

### Stability assays

The thermal stability of the antimicrobial compounds was analyzed by incubating 2 ml of the culture supernatant and the isopropanol cell wash extract at 37°C, 45°C, 65°C, 80°C and 100°C for 240 min. The stability of the antimicrobial activities against the treatment with proteases was determined by adding 10 mg/ml of proteinase K (Sigma-Aldrich, Taufkirchen, Germany), pronase E (Merck, Darmstadt, Germany) α-chymotrypsin (Sigma-Aldrich, Taufkirchen, Germany) and trypsin (Serva, Feinbiochemica, Heidelberg) to the cell-free supernatant and the isopropanol cell wash extract after evaporation of isopropanol and incubating at 37°C for 240 min. The influence of pH on the antimicrobial activity was examined by adjusting the pH to 1.5, 3, 9 and 13 and incubating for 30 minutes at room temperature. The residual antimicrobial activities were then determined in agar well diffusion assays against *M. luteus* ATCC 4698.

### Mass spectrometry analysis

The mass spectrometry analysis of the peptide preparations was performed using a MALDI-TOF mass spectrometer (Bruker Biflex, Bruker Daltonics, Bremen, Germany). Aliquots of 1 µl of the isopropanol cell wash extracts were mixed with 2 µl matrix (α-cyano-4-hydroxycinnamic acid in acetonitrile: 0.1% TFA in water, 1∶3). For MALDI-TOF analysis of the active HPLC fractions 20 µl of each fraction were concentrated 1∶10 using a rotational Vacuum Concentrator (RVC 2–18, Christ, Osterode, Germany). The samples were spotted onto the MALDI target and dried in air. Mass spectra were measured in positive ion mode in the range of 1500 to 4000 Da and analyzed by Flexanalysis 2.0 (Bruker Daltonics).

### Bioinformatic tools

The GC content was determined on the Genomatix homepage using the GEMS launcher (http://www.genomatix.de/cgi-bin/tools/tools.pl). The potential chymotrypsin cleavage site in Licβ was observed using the Expasy PeptideCutter Tool (http://www.expasy.ch/tools/peptidecutter/). Transmembrane sequences were identified using the TMHMM web server v. 2.0. (http://www.cbs.dtu.dk/services/TMHMM/). Blasts were performed at the NCBI nucleotide website (http://blast.ncbi.nlm.nih.gov/Blast.cgi). Subcellular localizations of the proteins were predicted using the online tool CELLO v.2.5 (subCELlular LOcalization predictor, http://cello.life.nctu.edu.tw/).
